# Metabolic Syndrome and Sarcopenia

**DOI:** 10.3390/nu13103519

**Published:** 2021-10-07

**Authors:** Hiroki Nishikawa, Akira Asai, Shinya Fukunishi, Shuhei Nishiguchi, Kazuhide Higuchi

**Affiliations:** 1 Second Department of Internal Medicine, Osaka Medical and Pharmaceutical University, Takatsuki 569-8686, Japan; in2108@osaka-med.ac.jp (A.A.); in2104@osaka-med.ac.jp (S.F.); kazuhide.higuchi@ompu.ac.jp (K.H.); 2 Premier Departmental Research of Medicine, Osaka Medical and Pharmaceutical University, Takatsuki 569-8686, Japan; 3 Kano General Hospital, Osaka 531-0041, Japan; nishiguchi@heartfull.or.jp

**Keywords:** metabolic syndrome, sarcopenia, mechanism, insulin resistance, sarcopenic obesity, outcome

## Abstract

Skeletal muscle is a major organ of insulin-induced glucose metabolism. In addition, loss of muscle mass is closely linked to insulin resistance (IR) and metabolic syndrome (Met-S). Skeletal muscle loss and accumulation of intramuscular fat are associated with a variety of pathologies through a combination of factors, including oxidative stress, inflammatory cytokines, mitochondrial dysfunction, IR, and inactivity. Sarcopenia, defined by a loss of muscle mass and a decline in muscle quality and muscle function, is common in the elderly and is also often seen in patients with acute or chronic muscle-wasting diseases. The relationship between Met-S and sarcopenia has been attracting a great deal of attention these days. Persistent inflammation, fat deposition, and IR are thought to play a complex role in the association between Met-S and sarcopenia. Met-S and sarcopenia adversely affect QOL and contribute to increased frailty, weakness, dependence, and morbidity and mortality. Patients with Met-S and sarcopenia at the same time have a higher risk of several adverse health events than those with either Met-S or sarcopenia. Met-S can also be associated with sarcopenic obesity. In this review, the relationship between Met-S and sarcopenia will be outlined from the viewpoints of molecular mechanism and clinical impact.

## 1. Introduction

According to the Japanese Ministry of Health, Labour and Welfare (MHLW)’s “Summary of the Vital Statistics Annual Report 2019 (approximate)”, the number of deaths in Japan was increasing in 2019. The aging of the population is thought to be a part of the reason. The leading cause of death was cancer at 27.3%, followed by heart disease (excluding hypertensive) at 15.0%, senility at 8.8%, and cerebrovascular disease at 7.7%, with diseases caused by lifestyle-related diseases such as obesity and hypertension accounting for a high percentage of deaths [1]. In Japan, obesity is defined as “an excessive accumulation of fat in adipose tissue, with a body mass index (BMI) of ≥25 kg/m^2^”, while the international classification of obesity defines obesity as a BMI of ≥30 kg/m^2^ [2].

Sarcopenia, defined by a loss of muscle mass and a decline in muscle quality and muscle function, is common in the elderly. The European Working Group on Sarcopenia in Older People (EWGSOP), the Asian Working Group on Sarcopenia (AWGS), and the Japanese Society of Hepatology (JSH) criteria for sarcopenia recommend the evaluation of both muscle mass and muscle strength as the definition of sarcopenia, but the phenotypes and specific reference values for the diagnosis of sarcopenia have not been completely standardized due to racial and other differences [3,4,5]. On the other hand, sarcopenia is also often seen in patients with acute or chronic muscle-wasting diseases, such as malignancies, pulmonary disease, heart failure, renal disease, neuromuscular disease, and liver disease (i.e., secondary sarcopenia) [5,6]. Skeletal muscle is a major organ of insulin-induced glucose metabolism. In addition, loss of muscle mass is closely linked to insulin resistance (IR) and metabolic syndrome (Met-S) [7]. In recent years, it has become clear that sarcopenia is closely related to Met-S, type 2 diabetes mellitus (T2-DM), and cardiovascular disease. Skeletal muscle loss and accumulation of intramuscular fat are responsible for impaired muscle contractile function and metabolic abnormalities and are associated with a variety of pathologies through a combination of factors, including oxidative stress, inflammatory cytokines, mitochondrial dysfunction, IR, and inactivity [8]. There is also growing interest in the clinical significance of skeletal muscle mass in chronic liver disease (CLD), cirrhosis, end-stage liver disease, nonalcoholic fatty liver disease (NAFLD), etc. [5,9]. The JSH sarcopenia assessment criteria are currently the only criteria for secondary sarcopenia specific to liver diseases around the world [5].

As mentioned above, there are various risk factors and etiologies for the development of sarcopenia, and one of the most notable topics is the relationship between Met-S and sarcopenia. Met-S refers to a condition in which there is excessive accumulation of visceral fat as well as elevated blood pressure, fasting hyperglycemia, and abnormal lipid levels, which increases the risk of T2-DM, cardiovascular disease, and cancer [10]. The Met-S prevalence was 34.1% among US adults in the National Health and Nutrition Examination Survey 1999–2006. According to the 2017 Japan National Health and Nutrition Examination Survey, the percentage of adults with strongly suspected Met-S was 27.8% in men and 12.9% in women. Including those who are considered to be in the pre-stage of Met-S (pre-Met-S) group, approximately one out of every two Japanese men is either in the pre-Met-S group or in the Met-S group. The MHLW has launched the “Healthy Japan 21 (Second Stage)” in April 2013, based on the final evaluation of “Healthy Japan 21” up to fiscal 2010 [11]. One of the basic measures for the promotion of the health of the people is to prevent the onset and worsening of lifestyle-related diseases. On the other hand, it is known that Japanese people are prone to T2-DM [12]. The higher disposition index (the value multiplying the insulin secretion capacity and the insulin sensitivity) in white US men than Japanese men can explain the lower susceptibility of white adults than Japanese adults to developing type 2 DM [12]. Japanese people have a lower insulin secretion capacity compared to Western people, and Japanese people are prone to developing T2-DM even if their BMI is low [13]. A cohort study of Japanese subjects reported that the risk of developing T2-DM was approximately three times higher in subjects with Met-S than in those without Met-S [14]. The presence of one or more features of Met-S in sarcopenic patients has also been shown to increase the risk of cardiovascular-related events, T2-DM, and other adverse events [15,16].

Lifestyle-related diseases are caused by a complex interplay of environmental factors and nutritional influences [10], and the number of researches on Met-S and sarcopenia has increased dramatically in the past few years, indicating the high level of attention paid by researchers. In this review, the relationship between Met-S and sarcopenia will be outlined from the viewpoints of molecular mechanism and clinical impact.

## 2. Mechanism for Sarcopenia Caused by Metabolic Syndrome

### 2.1. IR and Sarcopenia in Patients with Met-S

One of the main causes of Met-S is increased IR, and it is inferred that Met-S and sarcopenia are closely related through IR [10,17]. In compensatory hyperinsulinemia due to IR, glycogenesis is poorly suppressed, protein degradation is accelerated, and protein synthesis is reduced [18]. Hyperinsulinemia caused by IR also increases the amount of myostatin, which acts to reduce skeletal muscle [19]. Skeletal muscle is the major organ where glucose uptake by glucose transporter 4 (GLUT4) takes place. Skeletal muscle is responsible for about 80% of glucose clearance [20,21]. In mice, muscle-specific knockout of GLUT4 results in severe IR and glucose intolerance [22]. IR due to reduced skeletal muscle mass increases lipolysis, releases free fatty acids (FFAs) from adipose tissue, and inhibits the growth hormone (GH)-insulin like growth factor 1 (IGF1) axis, which promotes skeletal muscle protein synthesis [7]. In addition, myofibers (especially type IIb myofibers) can ameliorate metabolic abnormalities by secreting proteins and myokines, and loss of skeletal muscle mass due to aging or underlying diseases can exacerbate abnormalities in glucose metabolism [23]. IR causes enhanced glycogenesis, increased expression of sterol regulatory-element-binding protein 1c (SREBP-1c, described later), inhibition of β-oxidation, an increased supply of FFAs, and altered triglyceride transport, resulting in the accumulation of triglycerides in skeletal muscle and liver [24]. It is also thought that low levels of persistent inflammation in T2-DM patients may be responsible for the disruption of muscle homeostasis. Patients with increased IR have been shown to be at higher risk for loss of lean body mass (LBM), and worse glycemic control has been correlated with decreased physical performance [25]. Low LBM has been found to increase the risk of T2-DM and IR. The prevalence of sarcopenia in patients with T2-DM patients has been estimated to be as high as 15% [26]. In a Korean epidemiological study, out of 3305 individuals with Met-S, 739 (22.4%) had sarcopenia [27]. On the other hand, a close correlation between elevated HbA1c and muscle mass loss in non-obese patients with T2-DM has been noted [28]. Sugimoto et al. reported that HbA1c levels were more closely related to muscle mass loss (odds ratio (OR) = 5.42) than to low grip strength (OR = 1.89) or walking speed (OR = 1.13) in T2-DM patients with a BMI <25 kg/m^2^ [28].

### 2.2. Adipose Tissue and Sarcopenia in Patients with Met-S

White adipose tissues store excess energy that the body cannot use up as triglycerides and are found in large numbers under the skin and around visceral organs [29]. Brown adipose tissues, on the other hand, work to reduce fat by burning fat and consuming energy. Brown adipocytes contain a large number of mitochondria, and the uncoupling proteins (UCPs) involved in heat production present in the mitochondria take up fatty acids isolated from white adipocytes and convert them into energy [29]. The reduction of brown adipocytes mass reduces the efficiency of heat production, induces excessive fat accumulation, and causes Met-S [30,31]. Brown adipocytes are responsible for fat-burning, and the number of brown adipocytes decreases after the age of 40 [30,31]. Brown adipocytes are closely related to the function of adrenaline. Adrenaline stimulates brown adipocytes to increase heart rate and blood pressure and to produce heat to maintain vital functions in cold environments [30,31]. When adrenaline binds to the receptor, hormone-sensitive lipase is activated, resulting in FFAs, which are oxidized and degraded to become substrates for heat production. FFAs activate UCP1 in the mitochondria of brown adipocytes, causing stimulation of the sympathetic nervous system and fever [30,31].

There is a close relationship between the increase in visceral adipose tissue and muscle atrophy. The expression of contractile muscle proteins is reduced in myotubes co-cultured with white adipocytes from obese individuals [32]. In obesity and Met-S, white adipocytes become hypertrophic and hyperplastic. These white adipocytes are also infiltrated by activated inflammatory macrophages and other immune cells. These are associated with increased production of systemic inflammatory molecules [33] and suppressed production of various adipokines such as leptin, an appetite-suppressing hormone, and command, which adversely affect tissues such as the hypothalamus, liver, pancreas, and skeletal muscle [34]. Altered secretion of adipokines leads to increased food intake, decreased energy use, and decreased insulin sensitivity in muscles via their action in the hypothalamus. Proinflammatory cytokines such as TNF-α, monocyte chemoattractant protein-1 (MCP-1), IL-6, and CRP, which affect IR, are produced by adipose tissue, and muscle protein synthesis is reduced, inducing Met-S and sarcopenic obesity [35].

### 2.3. Persistent Inflammation

**Figure 1:**
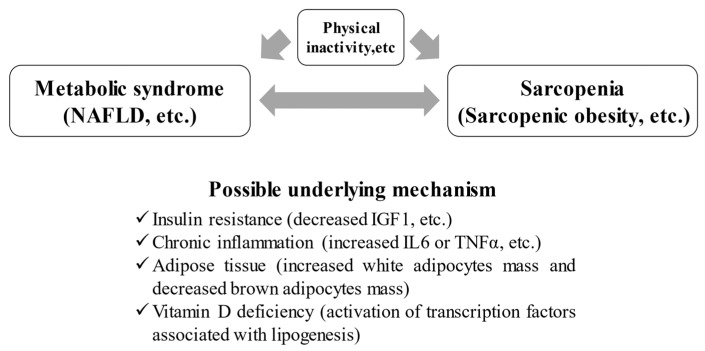
Correlation between metabolic syndrome and sarcopenia. NAFLD; nonalcoholic fatty liver disease, IGF; insulin-like growth factor.

Persistent inflammation, a factor in the pathogenesis of Met-S, is also closely related to sarcopenia. IL-6 is an inflammatory cytokine that can be elevated in numerous inflammatory diseases, and its effects on skeletal muscle have been studied. In one animal study, low concentrations of IL-6 were injected into the muscles of mice to determine the direct effects on skeletal muscle. In this study, the muscles exposed to IL-6 atrophied due to protein catabolism [36]. Increases in proinflammatory cytokines such as IL-6, CRP, and TNF-α have also been shown to adversely affect both muscle mass and function [37,38]. Inflammation, fat deposition, and IR are thought to play a complex role in the association between Met-S and sarcopenia (Figure 1). Because obese people have a higher percentage of visceral adipose tissue and more adipocytes that secrete large amounts of inflammatory cytokines, the muscle tissue of patients with Met-S is in a constant state of inflammation, elevating the risk of muscle atrophy [39]. In addition, obesity causes inhibition of muscle protein synthesis in skeletal muscle due to the accumulation of ectopic lipids in skeletal muscle (i.e., myosteatosis). This can increase the risk of IR in skeletal muscle [32]. A recent meta-analysis reported that myosteatosis is closely related to the prognosis in patients with cancer, and evaluation for muscle density is important as a marker [40].

### 2.4. Met-S, Sarcopenia, and Vitamins

Although epidemiological reports on vitamin D have previously shown that it is effective in preventing diseases such as Met-S and cancer, the mechanism of this effect was unclear [41,42]. Asano et al. focused on the SREBP-1c, a transcription factor that is one of the most important factors for lipid synthesis, and newly discovered that vitamin D metabolites regulate the activity of SREBP-1c [43]. A meta-analysis of studies on BMI and vitamin D reported that a 10% increase in BMI was associated with a 4% decrease in serum vitamin D levels [44]. It has been reported that an inverse trend was found between serum 25-hydroxyvitamin D level and Met-S (*p* = 0.051), which is thought to be due to the promotion of insulin secretion and reduction of fat synthesis by vitamin D intake [45]. It has also been shown that there are receptors for vitamin D in many organs throughout the body and that they are regulated by vitamin D [46]. On the other hand, vitamin D is fat-soluble and plays numerous important roles in skeletal muscle, such as maintaining muscle contractile excitability via intracellular calcium, proliferation and differentiation of skeletal muscle stem cells, and maintenance of muscle function; therefore, vitamin D is an essential hormone for the regulation of skeletal muscle function [47,48]. Vitamin D deficiency is closely related not only to Met-S but also to the development of sarcopenia (Figure 1) [47].

## 3. Met-S and Sarcopenia

### 3.1. Clinical Evidence of Met-S and Sarcopenia

A comparison of diagnostic criteria for Met-S between overseas countries (joint statement [49]) and Japan is shown in Table 1. Met-S and sarcopenia adversely affect QOL and contribute to increased frailty, weakness, dependence, and morbidity and mortality [50]. Furthermore, loss of muscle mass is associated with decreased survival in critically ill patients [51]. Patients with Met-S and sarcopenia at the same time have a higher risk of several health events than those with either Met-S or sarcopenia [16].

In a cross-sectional study of 533 Japanese women, the combination of Met-S and low muscle mass was shown to increase the risk of T2-DM [15]. A meta-analysis of 13 cross-sectional studies of 35,581 middle-aged and older non-obese adults showed that the prevalence of Met-S in those with sarcopenia was 36.45%, and there was a positive correlation between Met-S and sarcopenia [52]. In Japanese people, the association between Met-S and sarcopenia has been studied in 1971 community-dwelling elderly (mean age = 73 years). The prevalence of sarcopenia was higher in those with Met-S, with an OR of 4.99 in men aged 65–74 years, and there was a particularly strong association between visceral obesity and sarcopenia [53]. Compared to a decrease in skeletal muscle index (SMI), an increase in SMI of 1% or more per year significantly decreased the risk of developing Met-S [52]. Decreased skeletal muscle mass can cause Met-S, and increased skeletal muscle mass can prevent Met-S. Similarly, Kim et al. reported in their large study (*n* = 13,620) that there was a strong correlation between sarcopenia and the frequency of Met-S, with a 56% reduction in the risk of Met-S for every 1 quartile increase in limb SMI (*p* < 0.001) [54]. In a retrospective observational study of 11639 adult Koreans, 20.1% developed Met-S during a 7-year observation period [55]. The higher baseline SMI group had a significant reduction in the incidence of Met-S compared to the lower baseline SMI group (OR = 0.61). The ORs of Met-S in the groups with 0–1% increase in SMI and with 1% or more increase in SMI were 0.87 and 0.67, respectively, suggesting that increased muscle mass strongly inhibits the development of Met-S [55]. Similarly, an inverse correlation between the chronological change of skeletal muscle mass and the risk of Met-S is reported [56]. Study subjects were categorized into four groups: group A (from normal SMI to normal SMI), group B (from decreased SMI to normal SMI), group C (from normal SMI to decreased SMI), and group D (from decreased SMI to decreased SMI). Group D had increased ORs of Met-S, while group B revealed decreased ORs of Met-S, hypertension, and T2-DM. Group B also showed decreased ORs for all five Met-S components. Despite the fact that group C did not reach statistical significance, increased body fat and glucose fluctuation were identified in group C. The transition direction toward lower SMI (group C and D) presented deterioration in metabolic indices, resulting in elevated ORs for Met-S, hypertension, and T2-DM. Performing adequate intervention may effectively avoid adverse events accompanying lower SMI [56]. On the other hand, there are reports that muscle weakness is associated with the development of Met-S [57]. In a cross-sectional study of 833 elderly people, when classified into four groups according to the presence of dynapenia (i.e., lower grip strength) and visceral obesity, the group with both dynapenia and visceral obesity had the highest risk of Met-S (OR = 12.39) [57]. There are several other reports that lower grip strength is closely related to the development of Met-S [58,59]. Grip strength is strongly correlated with overall muscle strength and is closely linked to prognosis [60]. In view of these reports, physical inactivity is a major risk factor for both Met-S and sarcopenia (Figure 1).

### 3.2. Impact of Physical Inactivity

As humans age, physical activity declines [3,4,5]. There is a close relationship between the amount of physical activity and mortality. A large study (*n* = 83,034) that has examined the relationship between daily physical activity and all-cause mortality, as well as mortality from cancer, heart disease, and cerebrovascular disease, has shown that people who are more physically active have a lower risk of death [61]. The results of four groups according to physical activity level showed that the risk of death decreased in both men and women in the maximum physical activity group [61]. Compared to the minimum physical activity group, the risk of all-cause mortality was 0.73 times lower for men and 0.61 times lower for women in the maximum physical activity group. The maximum physical activity group of men had a 0.80-fold lower risk of cancer mortality and a 0.72-fold lower risk of heart disease-related mortality. The maximum physical activity group of women had a 0.69-fold lower risk of cancer mortality [61].

## 4. Sarcopenic Obesity and Met-S

A positive correlation between sarcopenia and obesity is pointed out [62]. A decrease in LBM leads to a decrease in physical activity, which in turn increases the risk of obesity. Accumulation of visceral adipose tissue causes sarcopenia by directly inhibiting the contractile proteins that skeletal muscle cells need to function properly [32]. The synergistic effect of adipose tissue and muscle tissue has given rise to a new and important concept in terms of health: sarcopenic obesity. This concept was first proposed in the work of Heber and colleagues [63], but a clearer definition was not developed until several years later. Sarcopenic obesity is defined as a condition involving a decrease in skeletal muscle mass, muscle weakness, and an increase in visceral adipose tissue, which simultaneously meets the following conditions: (1) muscle mass less than or equal to two standard deviations of the mean for young adults and (2) a body fat percentage greater than or equal to the median [64]. However, for reasons such as differences in body size among races, there is no consensus on the calculation method, and the reference values vary [65,66]. Furthermore, sarcopenia obesity is poorly clinically defined [16].

Subjects with sarcopenic obesity have an increased risk of developing physical dysfunction compared to subjects with obesity or sarcopenia alone [64,67]. The association between Met-S and sarcopenic obesity has been investigated in Taiwanese residents (mean age = 63.6 years). Compared with healthy subjects, the OR of Met-S was 11.59 for sarcopenic obesity and 1.98 for sarcopenia, and the risk for Met-S was significantly higher in sarcopenic obesity [68]. Depending on the combination of sarcopenia and obesity, four groups can be created: non-sarcopenia with non-obesity (nS-nO), non-sarcopenia with obesity (nS-O), sarcopenia with non-obesity (S-nO), and sarcopenia with obesity (S-O), and the incidence of Met-S-related diseases can be compared in each group. Previous studies reported that: (1) the prevalence of dyslipidemia is higher in S-O than in S-nO and nS-O [69]; (2) the prevalence of hypertension is 1.5 times higher in S-nO, 2.08 times higher in nS-O, and 3.0 times higher in S-O than in nS-nO [70]; (3) the prevalence of Met-S is 1.98 times higher in S-nO, 7.53 times higher in nS-O, and 11.59 times higher in S-O than in nS-nO [68]; (4) S-O is more common in women and those with a higher fasting glucose and higher homeostasis model assessment of IR (HOMA-IR) [71]; (5) mortality is 4.2 times more severe in S-O than in nS-O in patients with solid cancers of the respiratory and gastrointestinal tracts [72]; (6) S-O has an earlier decline in instrumental ADL than nS-nO [73]; and (7) S-O is most associated with disability [74]. On the other hand, many diabetic patients with a BMI <25 kg/m^2^ have also been found to have significant muscle adiposity. A Japanese study reported that even in people with a BMI between 23 and 25 kg/m^2^, the presence of any one of hyperglycemia, dyslipidemia, or hypertension reduces insulin function in muscle almost as much as in obese people [75]. Patatin-like phospholipase domain containing 3 (PNPLA3) gene polymorphisms (GG) can be involved as the cause [76]. GG is closely associated with the development or progression of NAFLD, and the percentage of Japanese with GG (25%) is higher than that of Western people [76]. It has also been shown that the combination of age-related muscle mass loss and Met-S may exacerbate arterial stiffness [15]. In Japanese women, pulse wave velocity, an index related to arterial stiffness, was higher in the group with both sarcopenia and Met-S compared to the Met-S alone or sarcopenia alone groups [15].

## 5. NAFLD, Met-S, and Sarcopenia

Globally, NAFLD is the fastest-growing metabolic liver disease and is also associated with a dramatic increase in the number of obesity and T2-DM patients, which has attracted much attention from researchers [77]. Younossi et al.’s meta-analysis showed that the estimated prevalence of NAFLD increased from 20.13% in 2000–2005, 23.75% in 2006–2010, and 26.80% in 2011–2015, and NAFLD has become a major socioeconomic burden [78]. Especially in the US, NAFLD is estimated to account for about 75% of CLDs in 2011 [79]. The incidence of NAFLD in Japan is about 30%, which is almost the same as the incidence of NAFLD in other Asian countries [80]. While the most common outcome of NAFLD is cardiovascular-related events, NAFLD encompasses a wide range of metabolic liver diseases, from simple fat deposition to nonalcoholic steatohepatitis (NASH), which can lead to cirrhosis and hepatocellular carcinoma [81]. Large international cohort studies have reported that the increase in the number of NAFLD patients is proportional to the increase in the number of Met-S patients [82], and a positive correlation has been shown between visceral fat, an important factor in Met-S, and hepatocellular fat deposition in NAFLD patients [83]. NAFLD, as well as Met-S, can cause sarcopenia (Figure 1) [75].

The pathogenesis of NAFLD/NASH and sarcopenia are interrelated through IR. Excessive triglycerides stored in adipocytes leak into the bloodstream in the form of FFAs. It is known that an increase in FFAs causes IR. In addition, FFAs leaked into the bloodstream are transported to the liver, causing NAFLD/NASH [84]. In NAFLD/NASH, IGF-1 is decreased, and muscle protein synthesis is reduced, leading to sarcopenia. Sarcopenia is also a risk for liver fibrosis development in NASH [85,86]. The degree of fibrosis development in NASH is closely related to prognosis [87]. On the other hand, vitamin D receptors are expressed in various cells, such as the liver and skeletal muscle, as described above. In addition, vitamin D regulates insulin receptor expression not only in pancreatic beta cells but also in peripheral tissues. Therefore, vitamin D has a significant impact on the pathogenesis of IR, Met-S, NAFLD, and sarcopenia [88,89]. In skeletal muscle, vitamin D plays an important role in myoblast proliferation and differentiation and skeletal muscle growth. Vitamin D deficiency in NAFLD exacerbates inflammation and promotes liver fibrosis [88,89].

A previous meta-analysis of five cross-sectional studies reported that sarcopenic patients had a 1.5-fold elevated risk of NAFLD compared with non-sarcopenic patients [90]. Similarly, a meta-analysis of six cross-sectional studies of 19,024 individuals showed that sarcopenic patients had a 1.3-fold elevated risk of NAFLD compared to non-sarcopenic patients [91]. A large, longitudinal, population-based cohort study (10,534 non-NAFLD subjects at baseline and 2631 NAFLD subjects at baseline) demonstrated that increased muscle mass effectively inhibits the development of NAFLD [92]. Reduced grip strength is not only associated with the development of NAFLD but also correlates with the severity of NAFLD [93]. It has also been reported that if grip strength is maintained, the incidence of NAFLD and Met-S is significantly reduced [94].

## 6. Interventions for Met-S-Associated Sarcopenia

Moderate physical activity for health maintenance has recently attracted attention around the world. Higher levels of physical activity have been reported to improve Met-S and sarcopenia [95,96,97]. Longitudinal studies have found that more active people have a lower incidence of Met-S and sarcopenia [95,96,97]. Exercise increases decorin (myokine associated with muscle fiber proliferation) and IGF-1, which have muscle-protein-synthesis-promoting effects, and conversely decreases myostatin, which has muscle-protein-synthesis-inhibiting effects [95]. A continuous exercise habit increases brown adipose tissue [98]. Brown adipose tissue also regulates myostatin secretion and is involved in the regulation of skeletal muscle mass and skeletal muscle function [99]. A well-balanced diet and regular exercise can help prevent Met-S-related sarcopenia. Leucine, which promotes muscle protein synthesis [100], sex hormones [101,102,103], myostatin inhibitors [104], and ACE inhibitors [105], which are involved in skeletal muscle synthesis, has been tested in clinical trials and reported positive results. On the other hand, a recent meta-analysis of vitamin D supplementation on muscle function did not show positive results [106]. Currently, there are no regulatory approved drugs for the treatment of Met-S-associated sarcopenia.

## 7. Closing Remarks

This article outlined the relationship between Met-S and sarcopenia, which has received much attention in recent years. Met-S and sarcopenia are interrelated through IR, adipose tissue, chronic inflammation, and vitamin D deficiency, etc. (Figure 1). Decreased muscle mass and strength are associated with the development of Met-S. Physical inactivity is a major risk factor for both Met-S and sarcopenia. Met-S can be associated with sarcopenic obesity (Figure 1). Hepatic fat mass in patients with NAFLD correlates well with visceral fat, an important factor in Met-S. Moderate exercise can improve Met-S and sarcopenia and contribute to the extension of a healthy life span. On the other hand, there is insufficient evidence for pharmacological interventions for Met-S-associated sarcopenia, and further clinical data collection is needed to confirm these results.

## Figures and Tables

**Table 1:** Comparison of diagnostic criteria for metabolic syndrome.

	Joint Statement [49]	Japanese Criteria
Definition	Three or more of the following items apply	Required item and at least two items in other items
WC	Each country adopts its own standards	<Required item> Male: ≥85 cm, female: ≥90 cm
TG	≥150 mg/dL or under treatment	≥150 mg/dL or under treatment
HDL cholesterol	<40 mg/dL or under treatment (Male) <50 mg/dL or under treatment (Female)	<40 mg/dL or under treatment
Blood pressure	≥130 mmHg (systolic) and/or ≥85 mmHg (diastolic) or under treatment	≥130 mmHg (systolic) and/or ≥85 mmHg (diastolic) or under treatment
FBG	≥100 mg/dL or under treatment	≥110 mg/dL or under treatment

WC; waist circumference, TG; triglyceride, FBG; fasting blood sugar.

## Data Availability

Data sharing is not applicable to this article.

## Abbreviations

MHLW: Ministry of Health, Labour and Welfare, BMI; body mass index, IR; insulin resistance, Met-S; metabolic syndrome, T2-DM; type 2 diabetes mellitus, CLD; chronic liver disease, NAFLD; nonalcoholic fatty liver disease, GLUT4; glucose transporter 4, FFA; free fatty acid, IGF1; insulin-like growth factor 1, SREBP-1c; sterol regulatory-element-binding protein 1c, LBM; lean body mass, OR; odds ratio, SMI; skeletal muscle index, NASH; nonalcoholic steatohepatitis.
